# Supplementary material for: Impact of change in household environment condition on morbidity in India: Evidence from longitudinal data
Source: PLoS One. 2021 Mar 3;16(3):e0247465. doi: 10.1371/journal.pone.0247465 (PMC7928466; doi:10.1371/journal.pone.0247465)
Supplement: Attachment — Submitted filename: Review Report of MRH on PONE-D-20_35348.pdf [file pone.0247465.s002.pdf]

Review Report on the manuscript reference number: PONE-D_2034358

Impact of Change in household environment condition on morbidity in India: Evidence from Longitudinal Data

This is an interesting paper that focuses on the 'Impact of Change in household environment condition on morbidity in India: Evidence from Longitudinal Data'' in the context of India. Overall, the paper has explored an important area. However, the author(s) should address the following weaknesses/comments before publication. The specific comments are:

- The authors sometimes used very long sentences in the abstract, introduction, discussion, and other places. The focus of the sentences is thereby imprecise. And the manuscript should check the semantic issues, especially in terms of using articles.
- The justification stated at the end of the introduction of the study does not match with the title. The authors mainly discussed sanitation facilities as part of the living environment. Given this, title modification/addition of more data on other indicators of the living environment is suggested in line with the study objectives. One way to solve the issuer is to define the 'living environment' first then add information on the indicators of the living environment.
- Reference(s) is needed in several places *(it is difficult to specify the sentence (s) as there is no line numbers and even no page number in the manuscripts).*
- The authors stated that: For the first time, this study investigates the causal relationships between household living environment and prevalence of short-term morbidities using data from the survey that followed the same households in 2004-05 and 2011-12. However, it is important to add information whether it is a first-time effort for the global context or in the context of India?
- The authors measured the household's hygiene condition (section Data Source). The inclusion criteria of such indicators would help the readers for replication of the study in another country context.
- Duplication of some information was found between the data source, and study design & study population.
- The definition HEC conditions are not clear from the author's description. Information about the estimated HEC Index's Alpha coefficient would also be added in the statistical analysis section. Are the reliability index values (Table 2) similar for used all 18 indicators?
- The study should explain the justification of using linear and multinomial logistic regressions (justification of using separate models) and of selecting the predictors precisely.
- Data (in %) for the sentences ''The average percentage of household members fell sick due to ASM was declined among the households who have experienced a better change in household environment conditions in 2011 compared to 2005' and 'Whereas average percentage of household members fall sick due to morbidity has been increased among the households who were lived in a clean environment condition in 2005 but they moved into poor household environment conditions in 2011'' are needed.
- The authors need to be consistent in terms of using decimal points throughout the text.
- The authors stated that morbidity has declined and increased between 2005 and 2011 between the groups. In these cases, the reference points (data in %) are missing. The stated sentences are:

  "Among the economically poor and non-poor households who were lived in poor household environment condition in 2005 but they turned into clean environment

condition in 2011, their morbidity has been declined 20.2% and 17.1% respectively. Whereas economically poor and non-poor households who were lived in clean environment condition in 2005 but they turned into poor environment condition in 2011, their morbidity was increased 23.6% and 21.1% respectively."

- The authors have described only those variables which were found significant in Table 6 and Table 7. However, it is suggested to add the non-significant variables also at the end of each table's description, with less focus.
- Please elaborate on caste groups (General, OBC SC, and ST) in the first place.
- The authors should incorporate references in several places of the discussion section such as:
    - The sentence in 2nd paragraph *(it is also suggested to split)*: ---due to implementation of the government schemes such as National Rural Drinking Water Programme, Total Sanitation Programme, Household Clean Energy Programme, and National Rural Health Mission, All these Schemes laid the foundation to improve household environment condition (HEC) under United Progressive Alliance (UPA) government, though, all these schemes successfully not achieved their aims due to poor operational management at ground level.
    - The sentence in 3rd paragraph The poor household environment condition persists due to open defecation, unimproved source of drinking water, unhygienic practices like not washing hand with soap after defecating, not cleaning toilet properly, cooking in unhygienic place and combustion of biomass for cooking.
- The conclusion section is not tightened enough. Thus it is suggested to revise this section linking with key findings and generate some specific policy recommendations.
- Since this is the first time effort as of the authors, limitations, and challenges that they faced are expected in the manuscript, which is completely missing. Moreover, information about ethical issues in terms of accessing and using the data set is expected
- Add description *** at the end of each table, where appropriate
- To what extend the linear regression model is suitable to explain the effects of predictors on outcome measure when the adjusted R-square is 0.02?

MRH
