# Supplementary material for: Impact of change in household environment condition on morbidity in India: Evidence from longitudinal data
Source: PLoS One. 2021 Mar 3;16(3):e0247465. doi: 10.1371/journal.pone.0247465 (PMC7928466; doi:10.1371/journal.pone.0247465)
Supplement: Attachment — Submitted filename: Remark PLOS.docx [file pone.0247465.s001.docx]

Author(s) attempt to examine the causal pathways between household environment condition and the morbidity profile of the households is highly appreciated especially given the current global emphasis on improving WASH indicators and consequently move towards healthier lives for all. India too has witnessed a surge of interest in promoting healthy living conditions with some flagship programs such as ‘Pradhan Mantri Ujjwala Yojana’ and ‘Swatch Bharat Abhiyan’ being initiated to promote hygienic and clean-living conditions. Thus, in view of such renewed national as well as international interest in improving household environment conditions and thus, a decrease in consequent morbidities, this paper provides important evidence to reiterate the association among these factors. The paper needs minor revisions mainly pertaining to grammatical errors. The detailed comments are mentioned below:

Remark 1: There are grammatical inconsistencies which need to be corrected since they impact the coherency of arguments used. The highlighted portions in few of the sentences illustrate the point, for instance:

“Because a large percentage of diarrheal morbidities is attributable to mortality due to lack safe drinking water”.

“Table 3 shows that the average percentage of members of households fall sick due to ASM (diarrhea, fever, and cough) by the change in household environment conditions with different socio-economic characteristics in India.”

“The chi squire statistics shown that the significance association change in members of households fall in sick due to any short-term morbidity

A thorough check for any grammatical errors and coherency needs to be done.

Remark 2: While discussing links between safe drinking water and morbidity, global figures regarding access to safe drinking water and sanitation has been cited by the authors but subsequent figures for India have not been mentioned. It is crucial to explain the existing condition of all WASH indicators and its trends in India since the paper discusses how changes in Household Environmental Condition affect the morbidity profile of households.

Remark 3: In the result section, author(s) have mentioned that “Whereas average percentage of household members fall sick due to morbidity has been increased among the households who were lived in a clean environment condition in 2005 but they moved into poor household environment conditions in 2011.” How does the movement of households into poor household condition in 2011 explain the morbidity profile of households in retrospect, i.e., in 2005??

Remark 4: In the result section, where state wise results are discussed, author(s) have mentioned that “The socio-economically underdeveloped states such as Punjab (35.5%), Chhattisgarh (29.5%), Uttar Pradesh (29.2%), Bihar (28.3%), and Uttaranchal (28.2%) have shown higher morbidity with respect to poor household environment condition”. Punjab is one of the most developed states in India, so it can hardly be categorised with the other states in the list in terms of their socio-economic profile.
