# Supplementary material for: Impact of change in household environment condition on morbidity in India: Evidence from longitudinal data
Source: PLoS One. 2021 Mar 3;16(3):e0247465. doi: 10.1371/journal.pone.0247465 (PMC7928466; doi:10.1371/journal.pone.0247465)
Supplement: Attachment — Submitted filename: Reply to comments and suggestions_PLOSE_ONE_.docx [file pone.0247465.s003.docx]

**Point by Point Replay to Reviewer’s Comments**

Manuscript ID No- PONE-D-20-35348: Impact of Change in household environment condition on morbidity in India: Evidence from Longitudinal Data

Date-11-12-2020.

To,

Editor-in-Chief

PLOSE ONE

Thank you for giving us the opportunity to revise our Manuscript titled “Impact of Change in household environment condition on morbidity in India: Evidence from Longitudinal Data” (ID No- PONE-D-20-35348). My co-authors and I are grateful to you and the reviewers for insightful and constructive suggestions. These valuable positive suggestions and comments have helped us improve our manuscript substantially. We believed that the revised version of the manuscript address the comments raised by reviewers. The summary of our revisions are organized in the order of your comments below.

**Reviewer –I**

Comment -1:  This is an important burning topic, effect of household environmental factor on morbidity. He has mentioned everything in the manuscript in planned way. Conclusion is clear and giving specific message to other researcher.

Author reply-I: Thank the referee for positive comment

**Reviewer –II**

Comment -II:  Tables: the p-values should not be 0.00 or 0.000. It should be <0.001. Some of the tables also miss the notes for star marks (*).

Authors’ reply-II: The suggestion is well taken and the same is incorporated in the manuscript

Comment -1II:  Second last paragraph of the results: “The lower morbidity among the ST households is probably due to under-reporting.” Please provide a reference or concrete logical argument for this sentence.

Authors’ reply-III: Normally the poorer sections of the population don’t consider/report the minor ailments as serious ones. People who are highly educated, richest and residing in developed regions report higher morbidity than their counterparts (less educated, poorer and residing in under developed regions). The suggestion is well taken and we have given concret logical argument for this statement in the manuscript.

**Reviewer –III**

Comment –I :  As the PLOS ONE is an international journal, the English version of any native schemes like Pradhan Mantri Awas Yojna or other such schemes should also be given. For example, the English version would be Prime Minister Housing Scheme.

Authors’ reply-I: The suggestion is well taken and it is incorporated in the revised manuscript.

Comment -II:  The authors have taken two outcome variables – first is the percentage of household members fall sick due to any short-term morbidity past one month preceding the survey period. And, the second one is recoded variable. I didn’t find use of first outcome variable in the manuscript. If it has been used, the author may clarify the same.

Authors’ reply-II: The comment is well taken. The first outcome variable is the percentage of household members fell sick due to any short term morbidity is a continuous variable. The main purpose of first outcome variable is to show the average percentage of household members fell sick due to any short term morbidity with respect change in household environment condition in different socioeconomic characteristics of the households. Whereas, it is not possible to show average percentage of household members fell sick due to any short term morbidity by second outcome variable with respect to change in household environment condition because the outcome variable is in categorical nature.

Comment -III:  The biggest disadvantage with the PCA for constructing composite index is that it gives normalized score which can not be used for over a period of time comparison (Means factor scores can be used for cross sectional analysis but not measuring progress over a period of time). Therefore, the classification of index score into four categories as (a) remaining poor in 2005 and 2011; (b) poor HEC in 2005 and non-poor in 2011; (c) non-poor HEC in 2005 and became poor in 2011; (d) remain non-poor HEC in 2005 and 2011 is not technically correct.

Authors’ Response-III: IHDS is a panel data which follows-up the same households, thus, our index doesn’t suffer from over a period of time comparison.

Comment -V:  Authors claimed that the reliability, validity and suitability estimates are given in the table 1, but I didn’t find any such value in table 1.

Authors’ reply-V: The comment is well taken. The reliability and validity was estimated for 18 household environment condition variables in table-2. The reliability and validity’s values are provided at the bottom of the table-2 for round-1 and round-2 of 18 variables of household environment condition.

 Comment -VI:  Table 1 and table 2 are not properly referred in the manuscript. They are not explained also.

Author replay-VI: The comment is well taken. Table 1 gives the description of the data and table 2 gives the variables used for the construction of HEC index. Normally these tables are not needed to be explained in detail in the manuscript.

Comment -VII:  Several studies have noted through their detailed empirical analysis that Muslims are socio-economically poor as compared to Hindus. The study also recognised this point but somehow claims that HEC of Muslims is much better than Hindus. The findings are contradictory in nature.

Authors’ reply-VII: Thank you for careful observation: Even though Muslim households are socioeconomically poor, their household environment condition is better-off than the Hindu households in terms of improved sanitation facilities and hygienic conditions as mentioned by previous studies( Coffey and Spears 2017; Coffey 2014). IHDS data of two rounds show that the Muslim household have more improved household environment condition than the Hindu households in India which I have analysed and submitted the paper to another journal for the consideration.

**Reviewers –IV, V and VII**

Comments-I: Reviewers IV, V and VII have suggested that the paper addresses a very vital question and thus should be considered for publishing after minor revisions. In current form however, the manuscript needs to be thoroughly checked for grammatical errors and sentence coherency. The entire manuscript needs serious editing for the English language. It needs an in-depth review for grammar, typographical and punctuation errors right from abstract to conclusion including tables (for instance table no. 3). The whole manuscript should be revisited for language corrections.

Authors’ reply-I. Thanks for the reviewers for their suggestion to improve the English language. We have improved the manuscript with respect to the English language.

Comment-II: In the abstract correct the spelling Swatch Bharat Mission. It should be Swachh Bharat Mission. The comment Background: Instead of sayings links, authors can consider saying association or impact in subheadings. The Last paragraph of background, authors claiming first time this study investigating. I suggest authors to revise the statement to “to our knowledge to date this is first study investigating the casual relationship”. The authors stated that: For the first time, this study investigates the causal relationships between household living environment and prevalence of short-term morbidities using data from the survey that followed the same households in 2004-05 and 2011-12. However, it is important to add information whether it is a first-time effort for the global context or in the context of India?

Author replay-II: Thank you for positive suggestions for improvement of the manuscript. We have incorporated the suggestions in the manuscript. We have revised the statement mentioning that “…… to jour knowledge to date this is the first study investigate the causal relation between any short term morbidity (Diarrhea, fever and cough) and household environment condition in the context of India”.

Comment-III: The study should explain the justification of using linear and multinomial logistic regressions (justification of using separate models) and of selecting the predictors precisely.

Author replay-III: The comment is well taken. The multivariate linear regression was used to assess the impact of change in household environment condition on morbidity. The outcome variable is percentage of household members fall sick due to any short term morbidity is a continuous variable. It shows the percentage of household members fell sick due to any short term morbidity is declined or increased/lower or higher with respect to change in household environment condition. It will not show the transition nature or change with respect to transition or change in household environment condition. Therefore, the multinomial logistic regression is used to assess the change in morbidity by change in household environment condition. In this context, the outcome variable shows that change or transition nature of morbidity is multinomial categories as remained same morbidity in both years in 2005 and 2011, higher morbidity in 2005 but lower morbidity in 2011, the lower morbidity in 2005 but higher morbidity in 2011 and remained same in both years in 2005 and 2011, with respect change in household environment condition. The morbidity variable is shown as transition in nature in this paper. We have incorporated the justification for using linear and multinomial logistic regressions in the paper.

Comment-IV: Data (in %) for the sentences ‘’The average percentage of household members fell sick due to ASM was declined among the households who have experienced a better change in household environment conditions in 2011 compared to 2005’ and ‘Whereas average percentage of household members fall sick due to morbidity has been increased among the households who were lived in a clean environment condition in 2005 but they moved into poor household environment conditions in 2011’’ are needed.

Author replay-IV: Wherever necessary, we have added the percentages in the sentences or in parentheses as per the suggestion of the referee.

Comment-V: The authors should incorporate references in several places of the discussion section such as: The sentence in 2nd paragraph (it is also suggested to split): ---due to implementation of the government schemes such as National Rural Drinking Water Programme, Total Sanitation Programme, Household Clean Energy Programme, and National Rural Health Mission, All these Schemes laid the foundation to improve household environment condition (HEC) under United Progressive Alliance (UPA) government, though, all these schemes successfully not achieved their aims due to poor operational management at ground level.

Author replay-V: We have included the reference as per the suggestion of the referee.

Comment-VI: Add description *** at the end of each table, where appropriate

Authors’ reply-VI: We have added the description as per the suggestion of the referee.

Comment-VII: To what extend the linear regression model is suitable to explain the effects of predictors on outcome measure when the adjusted R-square is 0.02?

Authors’ Response-VII: Adjusted R-square is too small because of controlling for too many variable in the model. However, lower adjusted r-square doesn’t qualify to completely reject the model.

Comment-VIII: It is surprising to note that the authors have applied ANOVA test among the variables while its interpretation is completely missing in the study.

Author replay-VIII: The comment is well taken and the same is incorporated in the revised manuscript.
